# Development and validation of clinical prediction models to distinguish influenza from other viruses causing acute respiratory infections in children and adults

**DOI:** 10.1371/journal.pone.0212050

**Published:** 2019-02-11

**Authors:** Danielle Vuichard-Gysin, Dominik Mertz, Eleanor Pullenayegum, Pardeep Singh, Marek Smieja, Mark Loeb

**Affiliations:** 1 Department for Health Research Methods, Evidence, and Impact, McMaster University, Hamilton, Canada; 2 Department of Internal Medicine, Cantonal Hospital Muensterlingen, Thurgau Hospital Group, Switzerland; 3 Department of Medicine, McMaster University, Hamilton, Ontario, Canada; 4 Michael G. DeGroote Institute for Infectious Diseases Research, McMaster University, Hamilton, Ontario, Canada; 5 Department of Pathology and Molecular Medicine, McMaster University, Hamilton, Ontario, Canada; 6 Child Health Evaluative Sciences, The Hospital for Sick Children, Toronto, Ontario, Canada; 7 St. Joseph’s Healthcare, Hamilton, Ontario, Canada; The University of Hong Kong, CHINA

## Abstract

Predictive models have been developed for influenza but have seldom been validated. Typically they have focused on patients meeting a definition of infection that includes fever. Less is known about how models perform when more symptoms are considered. We, therefore, aimed to create and internally validate predictive scores of acute respiratory infection (ARI) symptoms to diagnose influenza virus infection as confirmed by polymerase chain reaction (PCR) from respiratory specimens. Data from a completed trial to study the indirect effect of influenza immunization in Hutterite communities were randomly split into two independent groups for model derivation and validation. We applied different multivariable modelling techniques and constructed Receiver Operating Characteristics (ROC) curves to determine predictive indexes at different cut-points. From 2008–2011, 3288 first seasonal ARI episodes and 321 (9.8%) influenza positive events occurred in 2202 individuals. In children up to 17 years, the significant predictors of influenza virus infection were fever, chills, and cough along with being of age 6 years and older. In adults, presence of chills and cough but not fever were highly specific for influenza virus infection (sensitivity 30%, specificity 96%). Performance of the models in the validation set was not significantly different. The predictors were consistently found to be significant irrespective of the multivariable technique. Symptomatic predictors of influenza virus infection vary between children and adults. The scores could assist clinicians in their test and treat decisions but the results need to be externally validated prior to application in clinical practice.

## Background

Upper respiratory tract viral infections pose a substantial burden to the healthcare system [1–3]. During influenza seasons the number of outpatient visits for influenza in the U.S are estimated to range from 4.2 to 16.7 million which constitutes only a fraction of all medically attended cases of influenza-like-illness [4]. The average annual direct and indirect medical costs have been estimated to be $3.2 billion and 8.0 billion US dollar, respectively [5]. Influenza can lead to complications including hospitalization and death [6]. Since anti-viral therapy is available for influenza, predicting influenza can have important healthcare benefits.

Antigen-based rapid influenza diagnostic tests render results within minutes, are inexpensive and simple to apply. They are used as point-of-care diagnostic tests and show reasonable performance for ruling in influenza infection. However, their value for test and treat decisions is still limited because of the rather low sensitivity requiring more expensive molecular assays to reliably exclude the diagnosis of influenza [7, 8]. For influenza negative test results, the US Centers for Disease Control and Prevention (CDC) algorithm to assist in clinical decision-making recommends to rely on clinical signs and symptoms as well as on epidemiological information to guide further management [9].

However, there are a wide variety of symptoms associated with acute respiratory infection (ARI) including fever, chills, headache, myalgia, cough, sore throat, hoarseness, stuffed or runny nose, and sinus pain [10]. Clinical predictors for influenza have been broadly investigated [11–16]. Some of the limitations include single season studies or early discontinuation of surveillance [15, 16], and spectrum bias due to fever as part of the inclusion criteria [12, 13, 15]. None of these studies applied any form of internal validation.

The diagnostic performance of studies using clinical signs and symptoms to diagnose influenza has been reviewed and concluded that no combination of symptoms can accurately diagnose influenza, except in elderly where fever plus cough had a significantly increased likelihood of influenza [17]. However, the included studies were heterogenous with respect to age groups, countries of origin and design which may have lowered precision. Current clinical case definitions as issued by the CDC or the World Health Organization (WHO) comprise fever and cough or sore throat, but recent studies indicate that they do not perform sufficiently well [18, 19]. Aiming to diagnose influenza virus infection with higher accuracy we developed and validated symptom-based predictive scores in a population with a broader spectrum of ARI symptoms and laboratory confirmed diagnosis of influenza and other respiratory virus infections. This could be of interest from an infection control perspective if transmission of influenza virus to a susceptible population at risk for influenza complications is a concern and precaution measures should be promptly implemented.

### Patients and methods

We conducted a secondary analysis of a prospective cluster randomized controlled trial where Canadian Hutterite children and adolescents were vaccinated with either inactivated trivalent influenza vaccine (ITIV) or hepatitis A vaccine as a control in order to determine indirect protection to vaccine non-recipients [20].

The population included children and adults of Canadian Hutterite communities in the provinces Alberta, Saskatchewan, and Manitoba. The study began in September 2008, extended over 3 influenza seasons, and ended in July 2011. Signs and symptoms of respiratory infection including body temperature were recorded on a daily checklist. A family representative, usually the mother, was responsible for filling in the checklist for all the family members. To ensure optimal accuracy of the reported symptoms only older children and young adults could get their own diary if they were willing to fill it out. Trained study nurses visited the study colonies two times weekly, checked the diaries for missing data, and completed them with the family representative if required. ARI was defined as having at least 2 of the following symptoms: chills, cough, ear ache, fatigue, fever (≥ 38.0 C), headache, muscles aches, runny nose, or sore throat. A nasopharyngeal swab was obtained by the study nurse in all individuals that fulfilled the criteria for ARI and had 2 or more signs that were new since the last visit.

The respiratory specimens were examined by real-time polymerase chain reaction (RT-PCR) for the presence of influenza viral RNA.

Written informed consent was obtained. Individuals aged 16 years and older were required to sign. For enrolled children aged 15 years and younger, a signature had to be provided on the consent form by a parent, official guardian or family representative. In addition, all children aged 7 to 15 years had to read and sign an assent form, that was written in a simpler language, in order to fully enroll in the study. The study was approved by the Hamilton Integrated Research Ethics Board.

### Statistical analyses

We subdivided the data into two datasets, one for children (0–17 years) and the other for adults (18 years and older). Each dataset was then randomly split into a derivation set used for model development and an independent validation set to test the derived model performance. A larger derivation group of approximately 66% was chosen to preserve the power when conducting multivariable analyses. Assuming that there would be fewer variables in the final model a validation group of 34% of the original dataset was deemed appropriate. For the purpose of this analysis, only the first seasonal ARI episode that occurred during the influenza surveillance period was considered.

We performed univariable logistic regression for the comparison of demographic and clinical factors between the influenza and non-influenza groups and tested the crude associations first in each season separately and then pooled over all three seasons. Variables of interest included sex, seasonal history of receipt of influenza vaccine, age (in children), and all symptoms suggestive of ARI. All variables but age in children were dichotomized as present or absent whilst age in children was categorized into 0–5 years and 6–17 years in accordance with previous studies [15, 21].

We generated multivariable predictive models for the presence of influenza versus non-influenza virus-related ARI in children and adults using logistic regression as our primary analysis. Variables significant (P < .01) in the univariable analysis were entered into the multivariable analysis. Sex in adults, and age category (0 to 5 years vs. 6 to 17 years) and sex in children were predefined for entering multivariable regression models irrespective of their significance. In addition, fever, cough, and sore throat were considered *a priori* as clinically important due to their wide application in case definitions. For building of multivariable regression models, we used forward stepwise selection. We further applied generalized estimating equations (GEE) using the variables derived from the logistic regression model to account for repeated ARI episodes in different seasons in the same individual. Only the final models were then applied to the validation set. For each final GEE model, we generated coefficient-based point scores by dividing all regression coefficients by the coefficient with the smallest absolute value and rounding up to the nearest integer as previously published [22]. We then constructed ROC curves from the scores for the prediction of influenza virus infection and determined different cut-points to find optimal combinations of sensitivities and specificities.

As a sensitivity analysis we generated classification trees. One major advantage of this technique is the relatively simple integration of complex interactions that are usually avoided in parametric models [23]. We entered all predictors of interest into the classification tree models and applied the Gini index to reduce node impurity [24]. Point scores do not allow for the integration of interaction effects; thus, we did not assign point scores to the final classification tree models as this would have negated the advantageous characteristic of classification trees. The ROC curves were therefore constructed from the predicted probabilities of the final classification tree models. In the absence of a score we chose the sensitivities of the final classification tree models close to the sensitivities at the cut-points of the scores in the GEE models and determined the corresponding specificities along the ROC curves constructed from the predicted probabilities. From the sensitivities and specificities in the classification tree models we derived the remaining predictive indices analogous to the indices of the GEE models. Finally, the performance of the two multivariable models was compared by visually inspecting the ROC curves. Accuracy was classified according to the magnitude of the area under the ROC curve (AUC): 0.90–1.00 (excellent), 0.80–0.89 (good), 0.70–0.79 (moderate), and < 0.70 (poor). All statistical analyses were performed using IBM SPSS for Windows version 23.0 [25] and R software version 3.3.0 [26].

## Results

**Fig 1:**
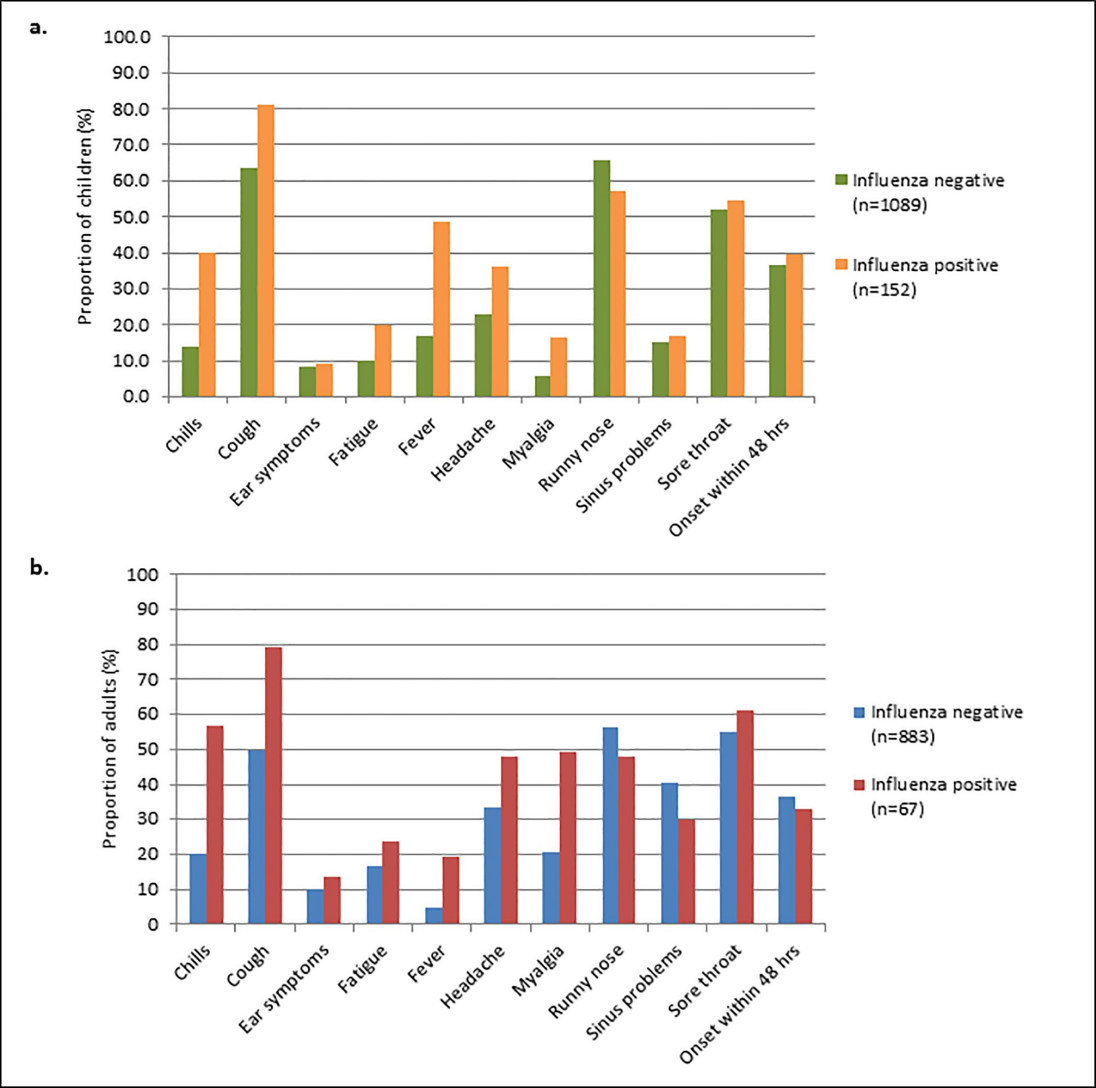
Symptoms of acute respiratory infection in laboratory confirmed influenza negative and positive children (a.) and adults (b.).

Between December 2008 and June 2011, 3332 first seasonal ARI episodes were observed. Of these, 44 episodes had to be excluded due to a missing outcome (either influenza diagnostic was not performed, or the result was indeterminate). There were eventually 3288 first seasonal ARI episodes in 2202 remaining subjects which accounted for 321 (9.8%) influenza A or B positive events. The frequencies and proportions of the various demographic predictors in the influenza positive and negative first seasonal episodes in the children and adults’ derivation sets pooled over all three seasons are depicted in Table 1. The proportion of influenza A virus infection and the distribution of influenza A subtypes was similar across the two age categories, children and adults. Overall, fever was less frequently reported in adults than in children and the proportion of children with laboratory confirmed influenza that reported fever was higher than the proportion of influenza positive adults with fever (48.7% versus 19.4%, respectively) (Fig 1A and 1B.).

**Table 1:** Characteristics of influenza positive and negative first seasonal episodes in the children and adult derivation set, influenza seasons 2008–2011. Values are indicated as numbers and percentages, n (%).

	Children (0–17 years)		Adults (18 years and older)	
	Influenza A or B		Influenza A or B	
	negative	positive	negative	positive
	n = 1089	n = 152	n = 883	n = 67
**Influenza A subtypes:**				
**A/H1N1 seasonal**	n.a.	15 (9.9)	n.a.	11 (16.4)
**A/H3N2**	n.a.	63 (41.4)	n.a.	31 (46.3)
**A/H1N1 pandemic**	n.a.	35 (23.0)	n.a.	12 (17.9)
**Female subjects**	642 (59.0)	82 (53.9)	631 (71.5)	39 (58.2)
**Influenza vaccine**	802 (73.6)	103 (67.8)	275 (31.1)	11 (16.4)
**Age category (6–17 years)**	685 (62.9)	104 (68.4)	n.a.	n.a.
**Age category (18–49 years)**	n.a.	n.a.	649 (73.5)	58 (86.6)
**Heart or lung disease**	56 (5.1)	5 (3.3)	77 (8.7)	4 (6.0)

n.a. not applicable

The results of the univariable and multivariable logistic regression analyses are shown in the appendix (S1 Table and S2 Table for children, S4 Table and S5 Table for adults).

### Prediction of influenza A or B infection in children

Presence of fever, chills, and cough, and being between 6 and 17 years old (versus < 6 years) were significant predictors for influenza A or B infection in the derivation set of the GEE model for children (Table 2). The corresponding estimates of the coefficients are listed in the appendix (S3 Table). When dividing the point score of the GEE model into different cut-points, it became evident that the presence of at least 3 predictors, which corresponds to a point score of 5 or more, was highly specific (85%) with a positive predictive value of 31%. Conversely, choosing a lower cut-point of 3, which corresponds to the presence of either fever or at least two other significant predictors, increased the sensitivity from 55% to 85% at the cost of a markedly lower specificity of 44% and a positive predictive value of only 16%. Assuming a prevalence of influenza A or B virus infection of 10%, the presence of fever alone or a point score of 3 would result in a children’s post-test probability of being influenza positive of only 9.9% considering the likelihood ratio of 1.5. Conversely, for the presence of at least 3 predictors (or a score of 5) a children’s post-test probability for being influenza A or B positive would be 29.1%. The performance of the GEE model in the derivation set was moderate (AUC 0.76; 95% CI 0.72–0.80). In the validation cohort the AUC was 0.70 (95% CI 0.63–0.77), the difference, however, was not statistically significant (p = .166).

**Table 2:** Summary of models for the prediction of influenza A/B virus infection in children (ages 0 up to 17 years).

Models	Predictors	Score	Set	AUC	p-value‡	Cut-point	Sens.	Spec.	PPV*	NPV*	pos. LR	neg. LR
				(95% CI)								
**GEE**	**Age 6–17 years**	1	**Deriv.**	0.76 (0.72–0.80)	0.166	**3**	85%	44%	16%	96%	1.5	0.34
	**Chills**	2				**5**	55%	85%	31%	94%	3.7	0.53
	**Cough**	2	**Valid.**	0.70 (0.63–0.77)		**3**	83%	40%	15%	95%	1.4	0.42
	**Fever**	3				**5**	50%	84%	28%	93%	3.1	0.59
**Classification tree**		n.a.	**Deriv.**	0.77 (0.73–0.81)	0.450	**n.a.**	82%	53%	18%	82%	1.7	0.34
	**Fever**											
	**Chills**						36%	92%	36%	92%	4.5	0.70
	**Cough**											
	**Runny nose**		**Valid.**	0.74 (0.67–0.80)		**n.a.**	80%	51%	17%	95%	1.6	0.39
	**Male sex**						34%	92%	34%	92%	4.3	0.72

AUC = area under the (receiver operating characteristic) curve; GEE = Generalized Estimating Equations; LR = likelihood ratio; n.a. = not applicable; NPV = negative predictive value; PPV = positive predictive value.

‡ z-test for the differences between two independent AUCs

* assuming a prevalence (~pre-test probability) of influenza virus A or B infection of 11%.

In the classification tree model fever was the most important predictor of influenza A or B followed by chills. Cough, runny nose and sex were additionally selected as important predictors. The classification tree model thereby unveiled potential interaction effects since these latter predictors, cough and sex, depended on the presence or absence of either chills and/or runny nose, respectively in the previous nodes (S1 Fig). No interaction was detected with age. Performance of the classification tree model was moderate in both the derivation (AUC 0.77; 95% CI 0.73–0.81) and the validation set (AUC 0.74; 95% CI 0.67–0.80), respectively, and the difference was not statistically significant (p = .450) (Table 2).

**Fig 2:**
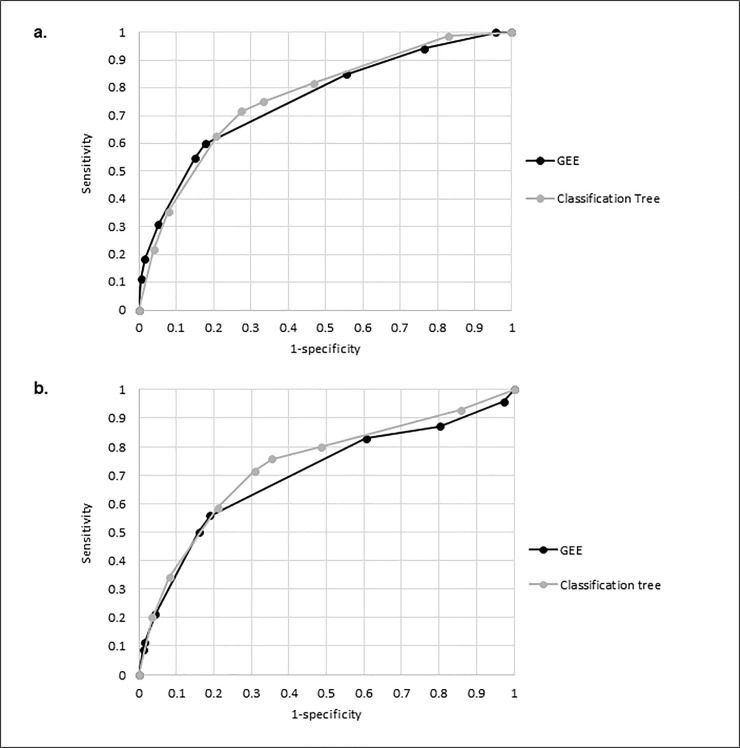
**Comparison of ROC curves among the two different models, GEE and classification tree, for the prediction of influenza A/B I children** (a. derivation set; b. validation set).

When comparing the two multivariable models by visualizing the ROC curves none of the predictive models showed clear superiority regarding performance (Fig 2A and 2B.).

### Prediction of influenza A or B infection in adults

In the GEE model the presence of chills, cough, and myalgia turned out to be significant predictors of influenza A or B infection in adults. According to the magnitude of their beta-coefficients, 2 points were assigned to each, chills and cough, and one point was assigned to myalgia for a total of 5 points (Table 3; S6 Table). A score of 4 or higher which corresponded to the presence of at least chills and cough yielded a specificity of 96% and a positive predictive value of 34% in the derivation set. The corresponding (positive) likelihood ratio of this score was 5.8. Assuming an adult’s pre-test probability of having influenza of 7%, the presence of chills and cough which is equal to a point score of 4 would have resulted in a post-test probability (risk) of 30% for being influenza A or B positive in this adult based on a (positive) likelihood ratio of 5.8. The model performance was moderate with an AUC of 0.78 (95% CI 0.72–0.85). Applying the GEE model to the validation cohort resulted in a similar performance as indicated by an AUC of 0.79 (95% CI 0.71–0.87; p = .866).

**Table 3:** Summary of models for the prediction of influenza A/B virus infection in adults.

Model	Predictors	Score	Set	AUC	p-value^‡^	Cut-point	Sens.	Spec.	PPV*	NPV*	pos. LR	neg. LR
				(95% CI)								
**GEE**	**Chills**	2	**Deriv.**	0.78 (0.72–0.85)	0.866	**2**	90%	38%	10%	98%	1.5	0.26
	**Cough**	2				**4**	30%	96%	34%	94%	5.8	0.59
	**Myalgia**	1										
			**Valid.**	0.79 (0.71–0.87)		**2**	94%	29%	9%	91%	1.3	0.21
						**4**	38%	92%	26%	95%	4.8	0.67
**Classification tree**	**Chills**	n.a.	**Deriv.**	0.80 (0.75–0.86)	0.410	**n.a.**	87%	53%	12%	98%	1.8	0.26
	**Cough**						46%	92%	30%	96%	5.8	0.59
	**Myalgia**											
	**Sinus problems**		**Valid**	0.75 (0.65–0.85)		**n.a.**	81%	53%	11%	97%	1.7	0.36
	**Sore throat**						37%	92%	26%	95%	4.6	0.68

AUC = area under the (receiver operating characteristic) curve; GEE = Generalized Estimating Equations; LR = likelihood ratio; n.a. = not applicable; NPV = negative predictive value; PPV = positive predictive value.

^‡^ z-test for the differences between two independent AUCs

* assuming a prevalence (~pre-test probability) of influenza virus A or B infection of 7%.

**Fig 3:**
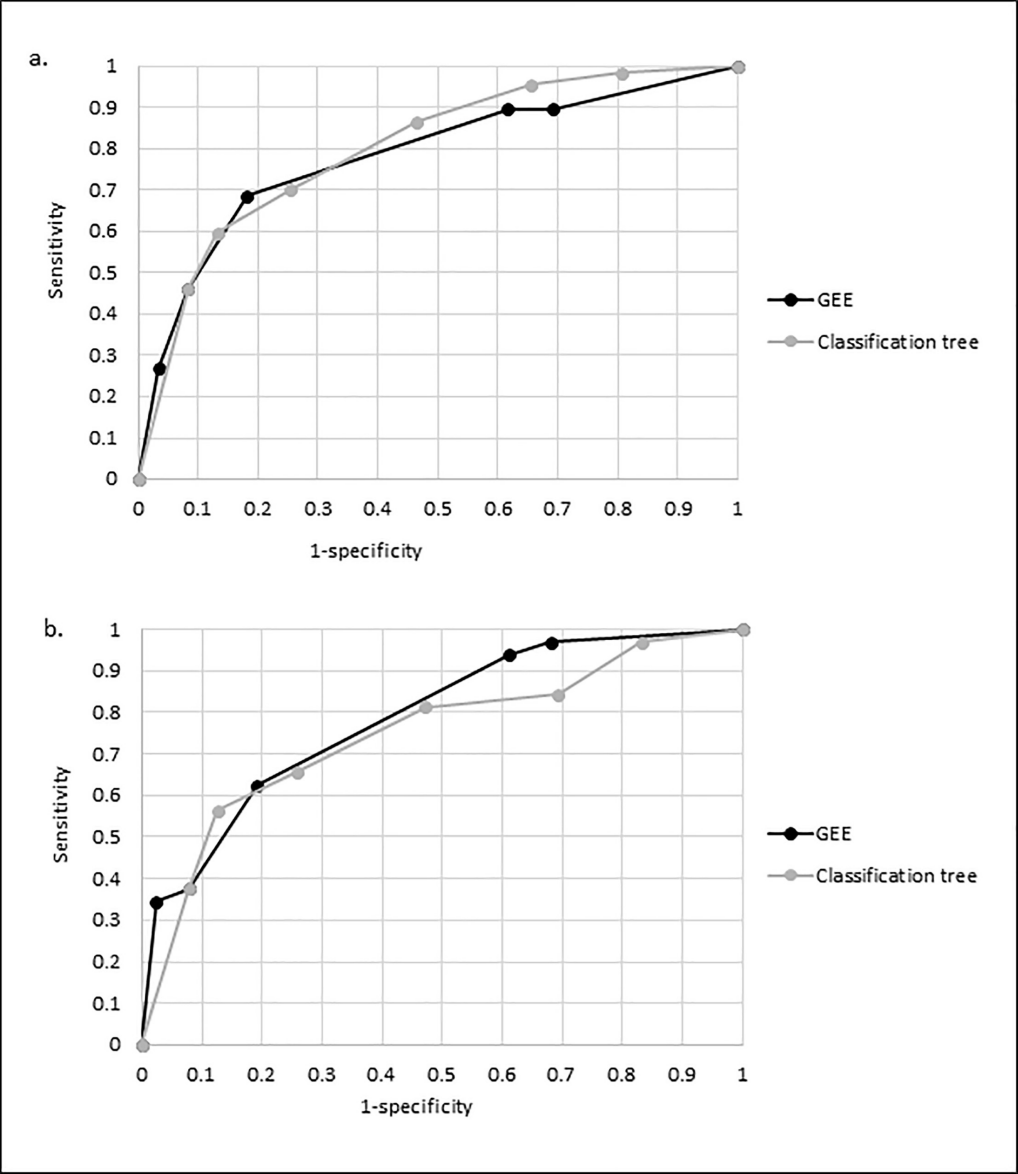
**Comparison of ROC curves among the two different models, GEE and classification tree, for the prediction of influenza A/B in adults** (a. derivation set; b. validation set).

Presence of chills, cough, and myalgia were also identified as significant predictors in the classification tree model. However, it also identified the presence of sinus problems and sore throat as significant variables for the prediction of influenza A or B infection (Table 3). It further discovered potential interaction effects between cough and myalgia, since myalgia had only a predictive value for the diagnosis of influenza A or B in the presence but not in the absence of cough (S2 Fig). The performance of the classification tree model in the derivation set was good with an AUC of 0.80 (95% CI 0.75–0.86), whereas performance was only moderate in the validation set (AUC 0.75; 95% CI 0.65–0.85). The difference, however, was not statistically significant (p = .410). Overall, visual comparison of the ROC curves across the models showed consistently moderate performance irrespective of the multivariable modelling technique and neither of the models clearly outperformed the other one (Fig 3A and 3B.).

## Discussion

Our results demonstrate robustly that in children fever, chills and cough are the key symptomatic predictors along with age from 6 to 17 years for the diagnosis of influenza virus infection whereas the essential predictors in adults were chills, cough, and myalgia but not fever. The almost equal performance of the two models in a second, independent cohort, underpins the validity of the results.

### How our results compare to previous studies

Our results are consistent with previous studies that showed an independent association of fever and cough with influenza virus infection in children [19, 21]. However, fever was eliminated from both models in the adult data set. From a pathophysiological perspective, it seems reasonable that fever would be significant in children but not in adults, since it indicates a strong innate immune response.

With that finding our study differs from prior studies that were conducted in one influenza season only and in patients predominately infected with influenza A/H3N2 [11, 13]. Reviewing clinical signs and symptoms in patients during the 2009 pandemic influenza A/H1N1 revealed that mild illness without fever occurred in 8–23% of infected patients whereas fever was the predominant symptom in hospitalized patients or was only a significant predictor of influenza A/H3N2 but not for influenza A/H1N1 [11, 27]. As a result of our less stringent inclusion criteria, we noted that the overall number of febrile episodes in the adult population (56 of 950 episodes or 5.9%) was rather low and that the majority (56 of 67 or 83.6%) of laboratory confirmed influenza episodes in our adult outpatient population were indeed afebrile. Together with the fact that influenza A/H3N2 was predominant only in season one and three but not in season two and that the majority of influenza episodes in adults were related to influenza subtypes other than influenza A/H3N2 may explain why fever was, overall, not selected as an important predictor. The total number of positive influenza episodes was, however, too small to adjust for influenza subtypes.

Overall, the differing results could be explained by the broader spectrum of symptoms in our study population and the larger variety of underlying pathogens which eventually facilitated the discriminative ability of these predictors to be detected. Since only a handful of participants required medical attention [28] we cannot rule out that the absence of fever in a majority of individuals was due to a milder course of influenza virus infection as has earlier been suggested [29]. On the other hand, more recent work has also shown that older adults being admitted to hospitals with laboratory confirmed influenza have lower body temperature than required for fulfilling the WHO definition of ILI [18].

In addition, sore throat was considered *a priori* in our multivariable models to be consistent with current ILI case definitions but was consecutively eliminated when adjusted for covariates. Although we have no biological explanation, our finding is in line with recent classification tree results from a large cohort of children and adults for the prediction of influenza virus infection [30]. These and our findings raise concern about the usefulness of the WHO’s and CDC’s case definitions either for surveillance reason or application in hospital settings. It seems possible, that a considerable proportion of symptomatic individuals in the community can transmit influenza unconsciously to susceptible individuals at risk of complications. This could be of relevance for healthcare settings were healthcare workers or visitors are potential sources of influenza virus transmission to susceptible patients.

### How our results could be applied in clinical practice

Although the common fever-and-cough rule validated by Boivin and colleagues [12] has been proven useful, our results show that it may not be comprehensive enough. With our study spanning over different seasons and in context with surveillance data confirming that influenza virus is circulating but without knowledge of the specific subtype(s), individuals without fever, especially adults, have to be considered to have influenza. With the lower and higher cut-points depending on the type and number of present predictors we demonstrated the considerable change in the magnitude of the predictive indexes. The main disadvantage of various rapid influenza diagnostic tests is their low sensitivity [7]. Provided that an increase in certainty would change management, the lower cut-point of our scores thereby could serve as threshold for complementary laboratory diagnosis whereas the higher cut-point could make any further diagnostic testing futile and could, therefore, potentially safe costs. The scores could also guide decisions on whether or not additional isolation precaution measures needed to be implemented before a laboratory test result would be available.

### Study limitations

Our study has some limitations. First, the low number of influenza positive events in children aged 0 to 5 years precluded us from analyzing this age group separately. Therefore, we cannot make a statement about any significant predictors for this specific age group. Second, the classification trees illustrated that interaction effects might be present which were not considered in our primary analysis. These results need to be interpreted with caution since the method is not as robust as the GEE model. Single tree models are unstable to small changes in the learning data which may result in high variability of the predictions [31]. We therefore regarded the classification tree models as sensitivity analyses to confirm the obtained results from the GEE models. In addition, the biological plausibility of such interactions remains uncertain and integration of these interaction effects did not increase the model’s accuracy. Thus, by adding to the models’ complexity they would rather challenge the models’ applicability in clinical practice.

Third, it is known that simple data-splitting is a less stringent validation method than e.g. splitting the groups with respect to time [32]. However, our aim was to generate a general prediction rule that holds true irrespective of the season and, thereby, ignores the potential influence of different circulating strains, an information that is not necessarily available.

Eventually, any prediction rule needs to be tested and validated in an external group of patients to ensure that it maintains its predictive power. The Canadian Hutterite community has been used as a model to understand herd immunity and the epidemiology of influenza for over 10 years. It is clearly a unique community. However, it does allow for a more in-depth exploration of epidemiologic aspects of how influenza spreads than most mainstream communities. Social structures even within mainstream society vary greatly and in fact the Hutterites might share more in common with other rural communities than other rural communities share with inner city neighbourhoods for example. In terms of the clinical predictors and testing in this study, we are not aware of any biological or even social factors that would prevent generalizability to other communities. Application of a new clinical prediction rule, even if externally validated, requires a population similar to the one the rule has been developed and validated. It is important to assure that the clinical prediction rule has relevant influence on decision-making. Neither of these requirements are fulfilled by this study, with the latter usually requiring an impact analysis ideally by means of a randomized controlled trial.

## Conclusion

Predictors of influenza virus infection vary between children and adults. Fever, chills, and cough along with age from 6 to 17 years were strong predictors of influenza virus infection in children whereas chills, cough and myalgia were found to be most predictive of influenza virus infection in adults. Common definitions for influenza-like illness such as the fever and cough rule cannot universally be applied. The derived scores would be simple to apply in clinical practice and could guide further laboratory testing, but their generalizability and impact on clinical decision-making remains to be determined.

## Supporting information

S1 Table Univariable analysis, influenza seasons 1–3, children derivation dataset. (DOCX)

S2 Table Multivariable logistic regression model for the prediction of influenza in the children derivation set. (DOCX)

S3 Table GEE model for the prediction of influenza in the children derivation set. (DOCX)

S4 Table Univariable analysis, influenza seasons 1–3, adult derivation dataset. (DOCX)

S5 Table Multivariable logistic regression model for the prediction of influenza in the adult derivation set. (DOCX)

S6 Table GEE model for the prediction of influenza A/B in the adult derivation set. (DOCX)

S1 Fig Classification trees for predicting influenza A/B virus infection in children (a. derivation set; b. validation set).
(DOCX)

S2 Fig Classification trees for predicting influenza A/B virus infection in adults (a. derivation set; b. validation set).
(DOCX)
