# Supplementary material for: Development and validation of clinical prediction models to distinguish influenza from other viruses causing acute respiratory infections in children and adults
Source: PLoS One. 2019 Feb 11;14(2):e0212050. doi: 10.1371/journal.pone.0212050 (PMC6370215; doi:10.1371/journal.pone.0212050)
Supplement: S1 Table — (DOCX) [file pone.0212050.s001.docx]

**S1 Table. Univariable analysis, influenza seasons 1-3, children derivation dataset.**

|  | **Influenza negative (n=1089)** | | **Influenza positive (n=152)** | | **p-value** | **OR** | **95% confidence interval** | |
| --- | --- | --- | --- | --- | --- | --- | --- | --- |
|  | n | % | n | % |  |  | lower | upper |
| Age < 6 years | 404 | 37.1 | 48 | 31.6 | 0.185 | 1.278 | 0.888 | 1.838 |
| Male subjects | 447 | 41.0 | 70 | 46.1 | 0.241 | 1.226 | 0.872 | 1.724 |
| Vaccine | 802 | 73.6 | 103 | 67.8 | 0.126 | 0.752 | 0.522 | 1.085 |
| Chills | 149 | 13.7 | 59 | 39.8 | <0.001 | 4.002 | 2.767 | 5.789 |
| Cough | 692 | 63.5 | 123 | 80.9 | <0.001 | 2.433 | 1.594 | 3.714 |
| Ear problems | 93 | 8.5 | 14 | 9.2 | 0.783 | 1.086 | 0.603 | 1.959 |
| Fatigue | 108 | 9.9 | 30 | 19.7 | <0.001 | 2.234 | 1.43 | 3.489 |
| Fever | 183 | 16.8 | 74 | 48.7 | <.0001 | 4.679 | 3.292 | 6.702 |
| Headache | 247 | 22.7 | 55 | 36.2 | <.0001 | 1.933 | 1.349 | 2.77 |
| Myalgia | 62 | 5.7 | 25 | 16.4 | <.0001 | 3.261 | 1.979 | 5.374 |
| Runny nose | 717 | 65.8 | 87 | 57.2 | 0.037 | 0.694 | 0.492 | 0.980 |
| Sinus problems | 164 | 15.1 | 26 | 17.1 | 0.512 | 1.164 | 0.739 | 1.832 |
| Sore throat | 569 | 52.2 | 83 | 54.6 | 0.586 | 1.099 | 0.782 | 1.546 |
| Acute onset | 400 | 36.7 | 60 | 39.5 | 0.512 | 1.12 | 0.79 | 1.59 |
