# Supplementary material for: Development and validation of clinical prediction models to distinguish influenza from other viruses causing acute respiratory infections in children and adults
Source: PLoS One. 2019 Feb 11;14(2):e0212050. doi: 10.1371/journal.pone.0212050 (PMC6370215; doi:10.1371/journal.pone.0212050)
Supplement: S2 Table — (DOCX) [file pone.0212050.s002.docx]

**S2 Table. Multivariable logistic regression model for the prediction of influenza in the children derivation set.**

| **variables** | | **Beta coeff.** | **Std. error** | **Wald** | **df** | **Sig.** | **OR** | **95% CI** | |
| --- | --- | --- | --- | --- | --- | --- | --- | --- | --- |
|  |  |  |  |  |  |  |  | **Lower** | **Upper** |
|  | Age_cat_child (1) | .566 | .200 | 8.040 | 1 | .005 | 1.762 | 1.191 | 2.605 |
|  | Chills(1) | 1.212 | .204 | 35.421 | 1 | .000 | 3.361 | 2.254 | 5.009 |
|  | Cough(1) | 1.125 | .212 | 28.127 | 1 | .000 | 3.079 | 2.032 | 4.666 |
|  | Fever(1) | 1.570 | .197 | 63.554 | 1 | .000 | 4.807 | 3.268 | 7.072 |
|  | constant | -3.881 | .267 | 211.729 | 1 | .000 | .021 |  |  |
