# Supplementary material for: Development and validation of clinical prediction models to distinguish influenza from other viruses causing acute respiratory infections in children and adults
Source: PLoS One. 2019 Feb 11;14(2):e0212050. doi: 10.1371/journal.pone.0212050 (PMC6370215; doi:10.1371/journal.pone.0212050)
Supplement: S3 Table — (DOCX) [file pone.0212050.s003.docx]

**S3 Table. GEE model for the prediction of influenza in the children derivation set.**

| **variables** | **Beta coeff.** | **Std. Error (SE)** | **p-value** | **OR** | **95% CI** | | **Influenza A/B score** |
| --- | --- | --- | --- | --- | --- | --- | --- |
|  |  |  |  |  | **lower** | **upper** |  |
| **Age cat. 6-17 years** | 0.559 | 0.204 | 0.006 | 1.7 | 1.17 | 2.61 | 1 |
| **Chills** | 1.202 | 0.210 | <.0001 | 3.3 | 2.21 | 5.02 | 2 |
| **Cough** | 1.127 | 0.219 | <.0001 | 3.1 | 2.01 | 4.74 | 2 |
| **Fever** | 1.555 | 0.202 | <.0001 | 4.7 | 3.18 | 7.04 | 3 |
| **Intercept** | -3.870 | 0.284 |  |  |  |  | - |
