# Supplementary material for: Development and validation of clinical prediction models to distinguish influenza from other viruses causing acute respiratory infections in children and adults
Source: PLoS One. 2019 Feb 11;14(2):e0212050. doi: 10.1371/journal.pone.0212050 (PMC6370215; doi:10.1371/journal.pone.0212050)
Supplement: S4 Table — (DOCX) [file pone.0212050.s004.docx]

**S4 Table. Univariable analysis, influenza seasons 1-3, adult derivation dataset.**

|  | **Influenza negative (n=883)** | | **Influenza positive (n=67)** | | **p-value** | **OR** | **95% confidence interval** | |
| --- | --- | --- | --- | --- | --- | --- | --- | --- |
|  | n | % | n | % |  |  | lower | upper |
| Female subjects | 631 | 71.5 | 39 | 58.2 | 0.022 | 1.79 | 1.083 | 2.985 |
| Vaccine | 607 | 68.8 | 56 | 83.6 | 0.011 | 0.43 | 0.224 | 0.840 |
| Chills | 179 | 20.3 | 38 | 56.7 | <0.001 | 5.15 | 3.094 | 8.585 |
| Cough | 439 | 49.7 | 53 | 79.1 | <0.001 | 3.83 | 2.094 | 7.001 |
| Ear symptoms | 87 | 9.9 | 9 | 13.4 | 0.349 | 1.42 | 0.680 | 2.964 |
| Fatigue | 148 | 16.8 | 16 | 23.9 | 0.137 | 1.56 | 0.865 | 2.807 |
| Fever | 43 | 4.9 | 13 | 19.4 | <0.001 | 4.70 | 2.386 | 9.270 |
| Headache | 297 | 33.6 | 32 | 47.8 | 0.019 | 1.80 | 1.095 | 2.972 |
| Myalgia | 182 | 20.6 | 33 | 49.3 | <0.001 | 3.74 | 2.254 | 6.200 |
| Runny nose | 497 | 56.3 | 32 | 47.8 | 0.176 | 0.71 | 0.432 | 1.168 |
| Sinus problems | 358 | 40.5 | 20 | 29.9 | 0.085 | 0.62 | 0.364 | 1.071 |
| Sore throat | 485 | 54.9 | 41 | 61.2 | 0.32 | 1.29 | 0.778 | 2.153 |
| Acute onset | 321 | 36.4 | 22 | 32.8 | 0.563 | 0.86 | 0.505 | 1.451 |
