# Supplementary material for: Development and validation of clinical prediction models to distinguish influenza from other viruses causing acute respiratory infections in children and adults
Source: PLoS One. 2019 Feb 11;14(2):e0212050. doi: 10.1371/journal.pone.0212050 (PMC6370215; doi:10.1371/journal.pone.0212050)
Supplement: S5 Table — (DOCX) [file pone.0212050.s005.docx]

**S5 Table. Multivariable logistic regression model for the prediction of influenza in the adult derivation set.**

| **variables** | | **Beta coeff.** | **SE** | **Wald** | **df** | **Sig.** | **OR** | **95% CI** | |
| --- | --- | --- | --- | --- | --- | --- | --- | --- | --- |
|  |  |  |  |  |  |  |  | **Lower** | **Upper** |
|  | **Chills(1)** | 1.465 | .285 | 26.481 | 1 | .000 | 4.328 | 2.477 | 7.561 |
|  | **Cough(1)** | 1.534 | .319 | 23.057 | 1 | .000 | 4.636 | 2.479 | 8.672 |
|  | **Myalgia(1)** | .968 | .286 | 11.442 | 1 | .001 | 2.633 | 1.503 | 4.615 |
|  | **constant** | -4.449 | .337 | 174.074 | 1 | .000 | .012 |  |  |
