# Supplementary material for: Development and validation of clinical prediction models to distinguish influenza from other viruses causing acute respiratory infections in children and adults
Source: PLoS One. 2019 Feb 11;14(2):e0212050. doi: 10.1371/journal.pone.0212050 (PMC6370215; doi:10.1371/journal.pone.0212050)
Supplement: S6 Table — (DOCX) [file pone.0212050.s006.docx]

**S6 Table.** **GEE model for the prediction of influenza A/B in the adult derivation set.**

|  | **Beta coeff.** | **SE** | **p-value** | **OR** | **95% CI**  **lower upper** | | **Influenza A/B score adults** |
| --- | --- | --- | --- | --- | --- | --- | --- |
| **Chills** | 1.465 | 0.287 | <.0001 | 4.3 | 2.47 | 7.60 | **2** |
| **Cough** | 1.535 | 0.328 | <.0001 | 4.6 | 2.44 | 8.83 | **2** |
| **Myalgia** | 0.967 | 0.287 | 0.001 | 2.6 | 1.50 | 4.62 | **1** |
| **Intercept** | -4.452 | 0.367 |  |  |  |  | - |
