# Supplementary material for: Development and validation of clinical prediction models to distinguish influenza from other viruses causing acute respiratory infections in children and adults
Source: PLoS One. 2019 Feb 11;14(2):e0212050. doi: 10.1371/journal.pone.0212050 (PMC6370215; doi:10.1371/journal.pone.0212050)
Supplement: S1 Fig — Classification trees for predicting influenza A/B virus infection in children (a. derivation set; b. validation set). (DOCX) [file pone.0212050.s007.docx]

**
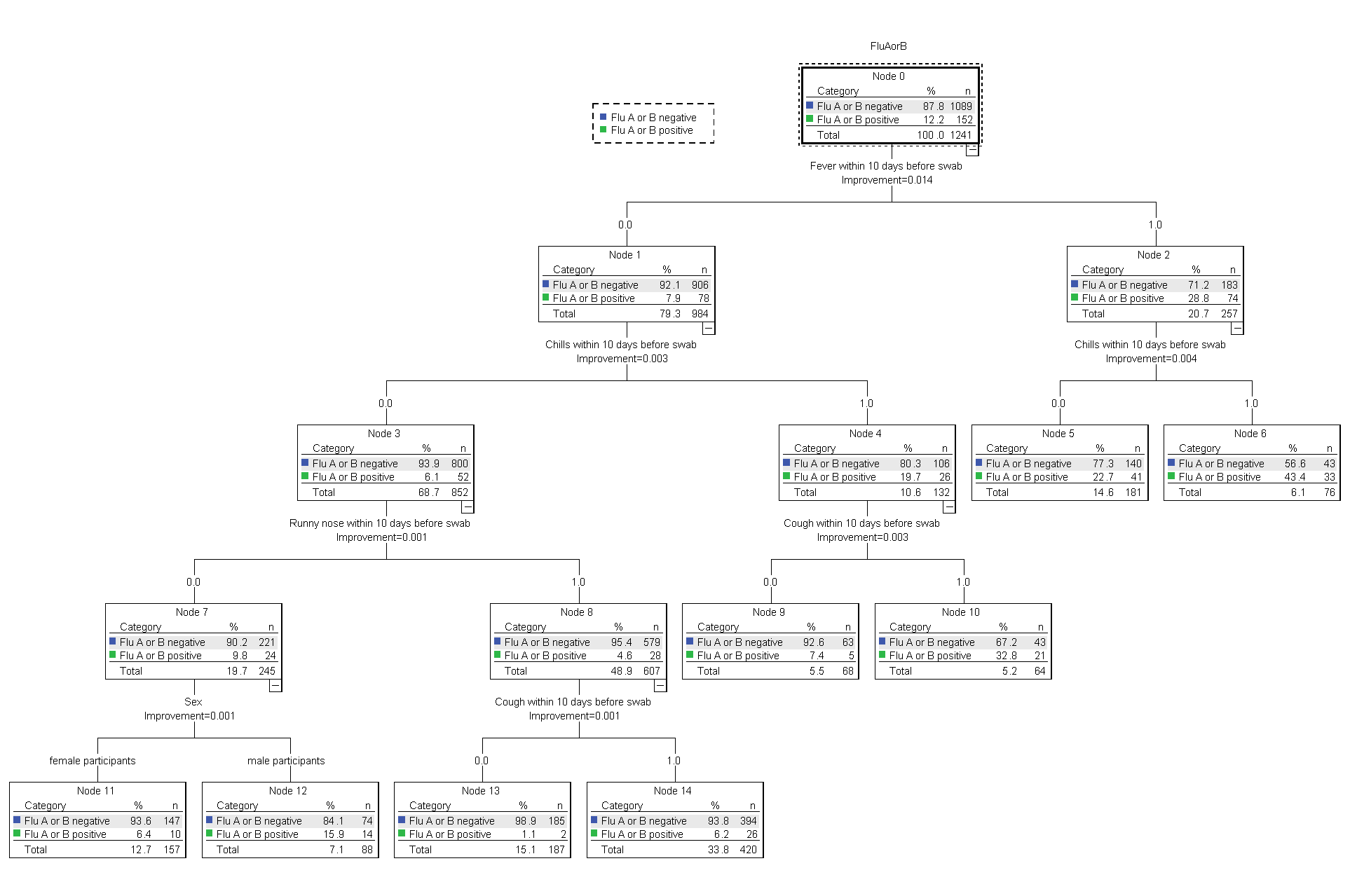
****S1 Fig.** **Classification trees for predicting influenza A/B virus infection in children** (a. derivation set; b. validation set).

a. Derivation set
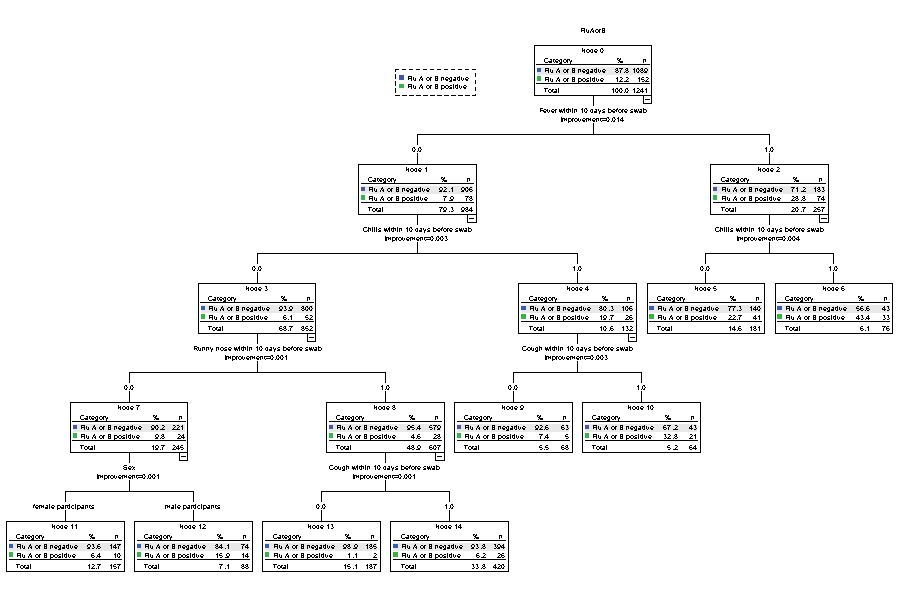


**
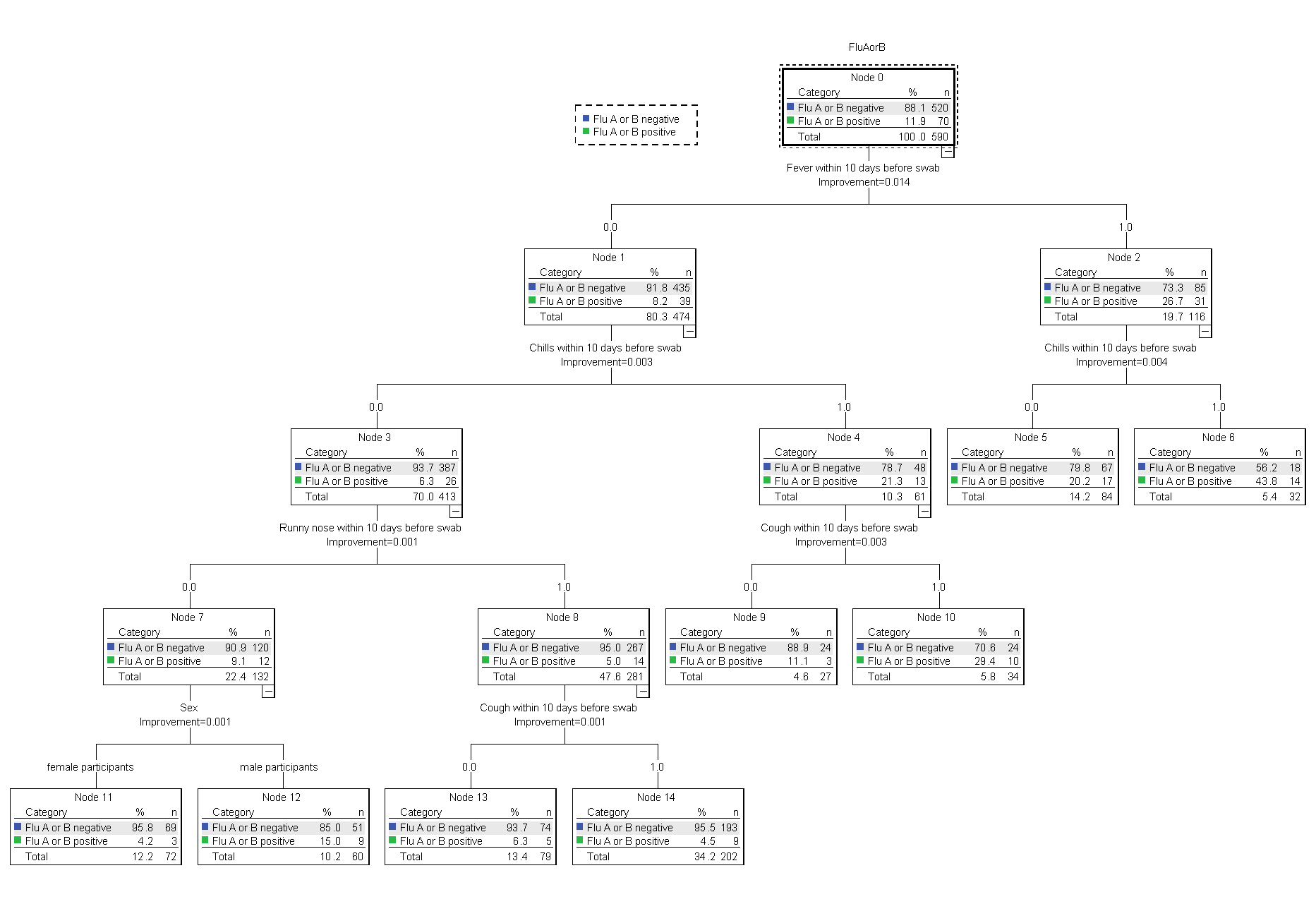
**

b. Validation set
